# Neurofilament light chain plasma levels in major depressive disorder: a brief research report

**DOI:** 10.3389/fpsyt.2024.1476248

**Published:** 2024-11-14

**Authors:** Carlos Fernández-Pereira, María de los Ángeles Fernández-Ceballos, José Manuel Olivares, José M. Prieto-González, Roberto Carlos Agís-Balboa

**Affiliations:** ^1^ Translational Neuroscience Group, Galicia Sur Health Research Institute (IIS-Galicia Sur), Área Sanitaria de Vigo-Hospital Álvaro Cunqueiro, SERGAS-UVIGO, CIBERSAM-ISCII, Vigo, Spain; ^2^ NeuroEpigenetics Lab, Health Research Institute of Santiago of Compostela (IDIS), Santiago University Hospital Complex, Santiago de Compostela, Spain; ^3^ Transalational Research in Neurological Diseases Group (ITEN), Health Research Institute of Santiago de Compostela (IDIS), Santiago University Complex, SERGAS-USC, Santiago de Compostela, Spain; ^4^ Neurology Service, Santiago University Hospital Complex, Santiago de Compostela, Spain

**Keywords:** neurofilament light chain, major depressive disorder, plasma, cognition, anhedonia; memory, psychiatry, biomarkers

## Abstract

**Introduction:**

Peripheral neurofilament light chain (NfL) reflect neuronal and axonal damage. Most studies have been focused on NfL cerebrospinal fluid measures since peripheral levels were difficult to detect. However, with recent advent of single molecule array (SIMOA) technology, NfL is now detectable peripherally at small concentrations (pg/ml). In neurodegenerative disorders, NfL peripheral levels have been found significantly elevated compared against psychiatric disorders. However, there is still controversy of whether NfL peripheral levels might be altered in psychiatric disorders like major depressive disorder (MDD) when compared against a normal population.

**Methods:**

We have measured NfL plasma levels by using single molecule array (SIMOA) technology in a Spanish small cohort of MDD patients (n = 15) and a HC group (n = 15). We have used subjective scales to estimate depression severity (HDRS), anhedonia (SAAS), the general cognitive state (MMSE) and episodic memory (FCSRT) in MDD patients.

**Results:**

We have not detected a significant alteration in NfL plasma levels in MDD patients when compared against the HC subjects (U = 97, *p-value* = 0.532). Moreover, we have not detected any significant correlation between NfL plasma levels with any subjective scales. The only parameter that significantly and positively correlated with NfL plasma levels was age in both MDD and HC.

**Discussion:**

Significant alteration in NfL plasma levels in MDD patients might reflect neurobiological changes behind the predisposition to develop future neurodegenerative disorders such as Alzheimer’s or Parkinson’s diseases for which depression represents a risk factor. However, whether there is an increase in NfL due to MDD regardless of the ageing process is still a matter of debate.

## 1 Introduction

Neurofilament light chain (NfL) is a subunit of neurofilaments that are found in neurons and axons, where they are major structural components of the axonal cytoskeleton (1). NfL is released into the extracellular space after the disintegration of the axonal membrane, caused by disruptive events such as neuronal death or axonal degeneration. From extracellular space, NfL reach the cerebrospinal fluid (CSF) where its concentration is measurable and may resemble axonal damage and degradation (2). Recent meta-analysis showed that NfL CSF levels are significantly elevated in many neurodegenerative disorders such as Alzheimer’s and Parkinson’s disease or multiple sclerosis (3). On the other hand, NfL CSF levels have been studied in primary psychiatric disorders (PPD), such as major depressive disorder (MDD), schizophrenia (SZ), or bipolar disorder (BD). First, no significant difference was found on NfL CSF levels between patients diagnosed with a psychiatric disorder and controls, whereas both were found significantly lower in contrast to neurodegenerative or neurological disorders, Moreover, NfL CSF levels have shown the best AUC (0.94, 95% IC 0.89-0.98) among other biomarkers such as Aβ1-42 or P-tau, selecting an optimal cut-off value of 1332 pg/ml with a sensitivity of 87% and a specificity of 90% to distinguish between neurodegenerative and psychiatric disorders (4). In case of MDD, NfL CSF levels have been found significantly elevated in a small sample of eleven elderly women with a previous history of MDD, regardless of not having developed dementia (5). Another study showed that NfL CSF levels were not statistically altered after six sessions of electroconvulsive therapy (ECT) in nine patients with a diagnosis of MDD, which may suggest that ECT treatment might not induce axonal damage in MDD patients (6). In other psychiatric disorders, no significant alteration was found in NfL CSF levels in a cohort of 100 patients with different diagnosis belonging to the SZ spectrum (7). In case of BD, NfL CSF levels have been found significantly elevated in euthymic BD patients and positively associated with the use of atypical antipsychotic treatment (8), whereas a longitudinal study made by the same research group yielded that higher CSF NfL levels have no significant association with a poor clinical outcome in BD patients after a 6-7 years follow-up (9). Interestingly, it was recently studied that NfL CSF levels were not altered in patients with dementia independently of having a history or not of depression (10).

*NfL also reaches the bloodstream with an around 40-fold lower concentration than in the CSF* (11), which initially made difficult the exploration of NfL levels as a potential peripheral marker. However, with the recent developments in ultrasensitive analytical methods like single molecule array (SIMOA) (12), NfL detection in peripheral tissues such as blood, serum or plasma has now become an achievable and relatively fast protocol (13). Therefore, SIMOA technology allows a less invasive approach than a lumbar punction to measure CSF levels and so the examination of NfL peripheral levels has drawn attention as a potential marker of acute axonal damage in different mental conditions (14). Initially, patients with a diagnosis of PPD have shown significant less NfL serum levels than patients with neurodegenerative diseases such as frontotemporal dementia (15) and frontotemporal lobar degeneration (FTLD). In their study, Katisko et al., set a serum cutoff value of 20 pg/ml to discriminate FTLD from PPD, with a discriminative sensitivity of 80% and a specificity of 85%, suggesting that PPD might not be entirely different or altered from a healthy control (HC) group (16).

During the last lustrum, several studies have investigated peripheral NfL levels in the context of PPD by using the SIMOA technology, including SZ spectrum (15, 17–20), BD (15, 21–24) and MDD (19, 22, 25–28). However, even before the development of fourth-generation immunoassays like SIMOA, some previous studies have also evaluated NfL peripheral levels by ELISA in SZ (29, 30), BD (29, 31) and MDD (24, 31–35).

To the best of our knowledge, the first study to apply the SIMOA technology in peripheral samples of patients with MDD found no significant alteration in NfL serum levels (26). On the other hand, ketamine-dependent MDD patients showed significantly higher NfL blood levels than HC (36). Similarly, NfL serum levels have been found significantly higher in MDD patients when compared against values taken from a normal population (19). In the same line, Chen et al., found significantly higher NfL plasma levels in patients with MDD. Curiously, NfL was significantly and positively associated with a poorer result in the executive function (24). Conversely, a recent study found no significant alteration in NfL plasma levels in a cohort of MDD patients who were mainly women (35). Lin et al., also proved that NfL levels were significantly elevated in MDD patients independently of being treated with ketamine or saline infusion (34). Huang et al., showed that NfL serum levels were significantly higher in patients with ketamine dependence, with or without a diagnosis with MDD, than a HC group, but not in patients with only a diagnosis of MDD (27).

Following this tendency, another study determined that NfL serum levels were not different between MDD patients, who were not following antidepressant treatment for at least two months before sample extraction, and HC subjects at baseline. However, NfL levels were found significantly increased after a three-month period (28). Contradictorily, Zhang et al., found significantly increased NF-L serum values in MDD patients in comparison to HC (37). Having these controversial results in mind, we aim to establish a cross-sectional study in a Spanish cohort in order to measure NfL plasma levels by using SIMOA technology comparing MDD patients and their symptomatology with a HC control group.

## 2 Method

### 2.1 MDD patients and HC subjects

We present a cross-sectional observational study that started in October 2017 with the recruitment process and ended in May 2024 with the experimental procedure. The recruitment process ended in March 2020 due to the beginning of COVID-19 in Spain. Samples were stored at -80°C until the acquisition of the SIMOA technology in May 2024. We recruited 15 patients who met the DSM-V diagnostic criteria for major depressive disorder (MDD group, n =15) at the Álvaro Cunqueiro Hospital (Vigo, Spain). We also recruited 15 voluntary participants without any history of previous psychiatric disorders who were included as a healthy control group (HC group, n = 15). HC were mainly conformed by volunteers, who were matched by age and sex distribution. First, we have their testimony of not having suffered from a mental illness. Second, they stated not having taken any psychiatric-related drug, such as antidepressants, antipsychotics or mood stabilizers. Third, corroborating these statements, there was no official record of psychiatric disorders or drug consumption in their clinical history.

The inclusion criteria included meeting the DSM-V major depressive disorder criteria, be of legal age (≥ 18 years), and delivery of the proper signed written consent. The exclusion criteria included additional comorbidities such as neurological pathologies. MDD patients were recruited at the hospital by the time they suffered from a MD episode, and so it was not possible to acknowledge whether MDD patients would have been following pharmacological treatment or not, since we only have their own testimony but not a controlled treatment regime.

All MDD patients and HC subjects that participated in this study had Spanish nationality. This research was carried out according to the Declaration of Helsinki. We have obtained written consent from all participants, or their legal tutors if was considered appropriate.

### 2.2 Pharmacological treatment

The scope of our study was to make a cross-sectional comparison between treated MDD patients and HC subjects. MDD patients were prescribed in their last clinical interview according to expertise clinical judgement (**Table 1**). We recruited them for our study based on our previously defined inclusion criteria (section 2.1). MDD patients had been taken multiple antidepressant classes, drug types and dosages, as it is normal on a daily clinical scenario.

**Table 1:** Last prescription of MDD patients before suffering from major depressive episode.

ID	SEX (F/M)	AGE (years)	Pharmacological Treatment					
			ADs	D (mg)	APs/MS	D (mg)	BZ	D (mg)
**MDD1**	F	27	Fluoxetine	20			Lorazepam	1
**MDD2**	M	23	Clomipramine	100	Aripiprazole	10	Zolpidem	10
**MDD3**	M	23	Venlafaxine	75			Clonazepam	1.5
**MDD4**	F	23	Desvenlafaxine	75				
**MDD5**	F	37	Escitalopram	40			Lorazepam	2
			Maprotiliine	120				
**MDD6**	F	35	Venlafaxine	75			Lormetazepam	2
**MDD7**	M	41	Venlafaxine	75			Lormetazepam	2
**MDD8**	M	43	Phenelzine	45	Lithium	800	Clonazepam	6
**MDD9**	F	52	Trazodone	100	Quetiapine	25	Tranxilium	10
**MDD10**	M	55	Trazodone	100	Amisulpride	60	Lorazepam	10
**MDD11**	M	50	Escitalopram	20			Alprazolam	2
			Amitriptyline	20				
**MDD12**	F	63	Escitalopram	40			Lorazepam	2
			Maprotiline	120				
**MDD13**	M	60	Venlafaxine	150	Aripiprazole	5		
			Duloxetine	60				
**MDD14**	M	67	Fluoxetine	100				
**MDD15**	F	63	Paroxetine	20				
			Clomipramine	75				

ADs, antidepressants; APs, antipsychotics; MS, Mood stabilizers; BZ, benzodiazepines or derivatives; D, dosage (mg).

On the other hand, MDD patients had no official record of prescription of non-steroidal anti-inflammatory drugs (NSAIDs). Time between last prescription and the inclusion of MDD patients in the study was 2 months on average, ranging from 4 to 10 weeks.

### 2.3 Sample extraction

Blood samples were extracted in the morning at the Álvaro Cunqueiro Hospital from antecubital portion of the arm in fasting conditions and stored in EDTA tubes. Plasma was then immediately separated employing a Ficoll-Paque gradient by centrifugation at 2000 rpm during 35 minutes. We then stored aliquots in the freezer (-80°C) until protein measurement at the Health Research Institute of Santiago de Compostela (IDIS).

### 2.4 Protein measurement

We defrost and centrifugated plasma samples at 10.000 RCF during 5 minutes according to the instructions of the manufacturer. Then, we pipetted 50 µl of plasma in each well of the 96-well plate and then we used the SIMOA^®^ NF-Light v2 Advantage Kit (QuanterixTM Corp, Billerca, MA 01821. Made in USA) to measure NfL plasma levels. We made two replicates of each sample with an intra-assay CV of 8.49 ± 6.69%. Measures were done by using the automatized SIMOA HD-X system.

### 2.5 Subjective scales

We measured the severity of depressive symptoms by using the Hamilton Depression Rating Scale (HDRS) (38). To measure anhedonia, we used the Self-Assessment Anhedonia Scale (SAAS) (39). The general cognitive state was evaluated with the Mini-Mental State Examination (MMSE) (40). On the other hand, we employed the Free and Cued Selective Reminding Test (FCSRT) to measure encoding memory (41). These scales were previously described elsewhere (42). Unluckily, we could not have access to cognitive tests in the HC group, since these tests were done only in MDD patients by expertise in their clinical evaluation.

### 2.6 Statistical analysis

The continuous parameters are presented as mean and standard deviation. We have inspected each variable with the Shapiro–Wilk test to verify whether the quantitative parameters (NfL plasma levels, subjective scales and age) could be adjusted to a Gaussian or Normal distribution (reported as: S-W (df) = F, p > 0.05) or not (p < 0.05). If they were found normally distributed and non-significant to the Levene’s test (p > 0.05), we then used the parametric Student’s t test (reported as: t (df) = F, p-value) to compare both distributions. If quantitative variables were not normally distributed or significant to the Levene´s test, we used the non-parametric Mann–Whitney’s test (reported as: U, p-value). Finally, we employed the Spearman’s correlation coefficient (reported as: r (df) = rs, p-value) to look for association between variables. We have also performed a linear regression equation between NfL levels and age. We made the formal statistical analysis using the software GraphPad Prism 7.05 version.

## 3 Results

### 3.1 General Data

Data from MDD patients and HC subjects are reported in **Table 1**. Both groups (HC, MDD) were not significantly different in terms of sex distribution (χ2 (1, N=30) = 0.133, p-value = 0.751), nor in mean age (t (30) = 0.09, p-value = 0.925).

### 3.2 Correlation between NfL plasma levels with age and sex distribution differences

**Figure 1:**
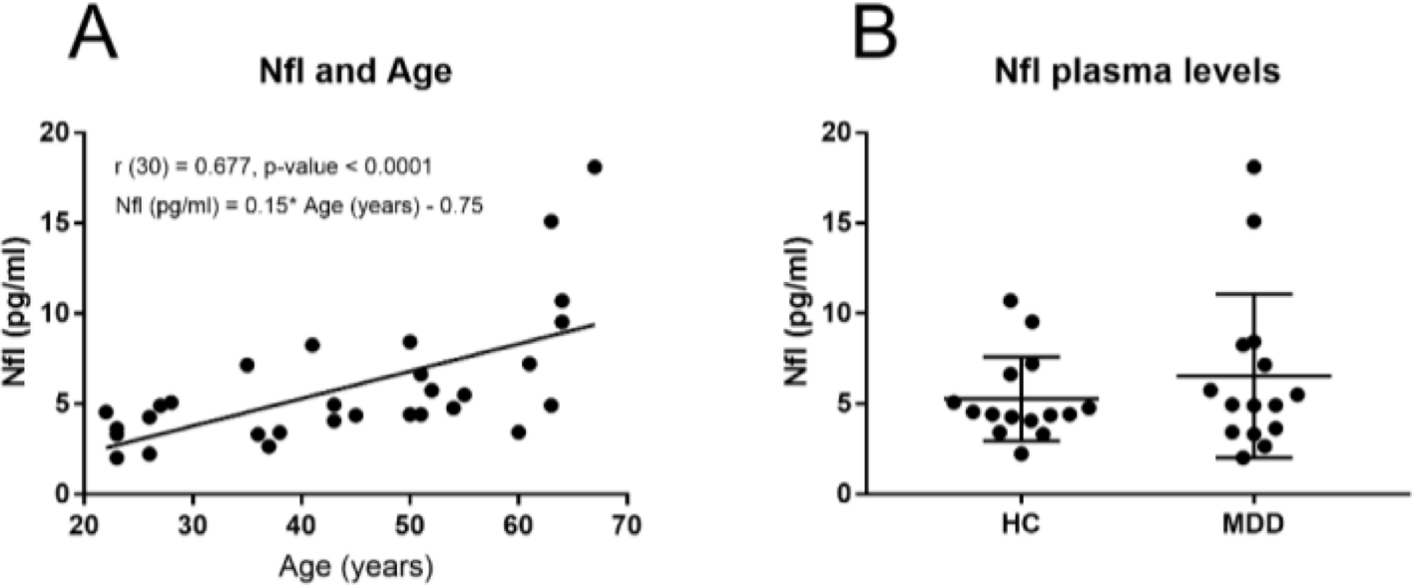
**(A)** Correlation and regression equation between NfL plasma levels (pg/ml) and Age (years) in the whole sample and, **(B)** NfL plasma levels in MDD patientes and HC subjects.

The NfL plasma levels were significantly and positively correlated with age in the whole cohort (r (30) = 0.657, p-value < 0.0001) and also in both the HC (r (15) = 0.740, p-value = 0.002) and the MDD group (r (14) = 0.608, p-value = 0.018). We then calculated a linear regression model to predict the effect of age on NfL plasma levels. The regression equation was statistically significant (F (1, 28) = 17.50, p-value = 0.0003). The R-square was 0.38 and the regression equation of the NfL plasma levels was 0.15 * age - 0.75 (pg/ml) and can be seen plotted in **Figure 1A**.

On the other hand, we did not find any significant difference in the NFL plasma levels between females (5.78 ± 3.23 pg/ml) and males (6.03 ± 4.03 pg/ml) in the whole cohort (U = 119.5, p-value = 0.992). These differences were not found significant either in the HC group (U = 28.5, p-value = 0.741) nor in the MDD group (U = 23, p-value = 0.613).

### 3.3 Comparison of NfL plasma levels between MDD patients and HC subjects

Despite the fact of the increased tendency in the NFL mean plasma levels (**Figure 1B**) observed in the MDD group (**Table 2**), the NFL plasma levels were not significantly different between the HC subjects and the MDD groups of patients (U = 97, p-value = 0.532).

**Table 2:** General data and NF-L plasma levels in HC and MDD group.

Variables	HC (n = 15)	MDD (n = 15)	p-value
Age (years)	43.93 ± 14.17	44.13 ± 15.76	0.925^1^
Sex (F/M)	7/8	8/7	0.751^2^
NfL (pg/ml)	5.27 ± 2.32	6.55 ± 4.53	0.532^3^
Female	4.84 ± 1.52	6.08 ± 4.35	0.955^3^
Male	5.65 ± 2.90	6.96 ± 4.94	0.536^3^

^1^Student’s T test was used. ^2^Chi-square Test ^3^Mann-Whitney Test was used. NfL, Neurofilament light chain plasma levels; HC, Healthy Control group; MDD, Major Depressive Disorder group.

On the other hand, no significant difference (U = 22, p-value = 0.536) was found between males in the MDD group (n = 8, 6.96 ± 4.94 pg/ml) and the HC group (n = 8, 5.65 ± 2.90 pg/ml). In the same line, no significant difference (U = 27, p-value = 0.955) was found between females in the MDD (n = 7, 6.08 ± 4.35 pg/ml) and the HC group (n = 7, 4.84 ± 1.52 pg/ml).

### 3.4 Correlation between NfL plasma levels and subjective scales in MDD patients

We have not found any significant correlation between the NfL plasma levels and the subjective scales (**Table 3**).

**Table 3:** Correlation between NfL plasma levels and subjective scales in MDD patients.

Subjective Scales	NfL (pg/ml)	
	r	p-value
SAAS	0.007	0.985
HDRS	-0.497	0.061
MMSE	-0.347	0.205
FCSRT_TFR	0.189	0.496
FCSRT_TR	-0.266	0.336
FCSRT_DFR	0.364	0.182
FCSRT_DTR	-0.052	0.854

Spearman’s correlation coefficient was used. NfL, Neurofilament light chain plasma levels; SAAS, Self-Assessment Anhedonia Scale; HDRS, Hamilton Depressive Rating Scale; MMSE, Mini-Mental State Examination; FCSRT, Free and Cued Selective Reminding Test; TFR, Total Free Recall; TR, Total Recall; DFR, Delayed Free Recall; DTR, Delayed Total Recall.

## 4 Discussion

In this brief report, we have measured NfL plasma levels by using the recently added SIMOA technology in a small Spanish cohort of MDD patients and HC subjects. We have not found significant alterations in the NfL levels in the MDD group compared to HC. Our findings are consistent with previous reports in the field (26–28, 35). However, some of these studies had to face similar limitations to ours. One of these studies employed a similar sample size of fifteen participants of both MDD patients and HC subjects before and after the effects of ECT therapy, leading to a non-significant elevation in NfL levels, suggesting no brain damage after ECT (26). On the other hand, Wallensten et al. measured NfL levels in thirty-one MDD patients who were mainly women (twenty-six), (35). We did not include sex as a potential confounding factor since no significant differences were found between males and females in our cohort. A recent meta-analysis evaluating NfL levels on MDD found no consensus about the influence of sex on NfL levels (43). Moreover, there is no clear effect of gender on peripheral NfL levels in HC when either age or BMI-corrected (44). On the other hand, ketamine-dependence has been widely studied when evaluating NfL peripheral levels in MDD (27, 34, 36). These studies lacked of a control group with a diagnosis of MDD but no ketamine as treatment. In this sense, Huang et al. proved that patients with only a diagnosis of MDD had no significant elevated NfL levels in contrast to a HC group (27). In the above-cited studies, MDD patients were under pharmacological treatment by the time samples were extracted. However, treatment does not seem to normalize NfL peripheral levels. Recently, it was determined that even MDD patients who were washed-out from treatment for at least two months before sampling, showed no alteration in NfL levels (28).

Conversely, other studies have shown that NfL peripheral levels are significantly elevated in MDD (19, 24, 31, 34, 36, 37). Nonetheless, there also may be some limitations regarding these studies. Bavato et al. compared NfL levels from MDD patients with a referenced normal range but lacked of an internal HC group with experimental measures of NfL (19). On the other hand, two above-cited studies found NfL levels significantly elevated in ketamine-dependent MDD patients (34, 36), but NfL significant elevation was also found in a group treated with just a saline infusion instead of ketamine (34). Therefore, whether the significant elevation in NfL peripheral levels is due to ketamine dependence or MDD may not be clear yet, but evidence points to a significant implication of ketamine treatment rather than MDD diagnosis. Zhang et al., showed significantly higher NfL levels in a small cohort composed of only four MDD patients and four HC subjects (37). In this sense, the study of Chen et al., might be the most complete one, with a sample size of forty on each group. However, as it was our case, treatment type, regime or dosage were not specified and so is difficult to distinguish whether the observed effects are a secondary effect from treatment or a intrinsic trait of MDD (24).

In spite of the controversial results found in the scientific literature, NfL peripheral levels seem to be associated with depressive symptomatology in elderly MDD patients (45, 46). In the present work, we have found a significant, strong and positive association between NfL plasma levels and age in the whole sample and in both the MDD and HC groups. Gudmundsson et al. measured elevated NfL levels in the CSF of elderly patients with a diagnosis of MDD (5). To some extent, elderly MDD patients might have a neurodegenerative dimension (13), since MDD is considered a risk factor for neurodegenerative disorders such as AD (47). Moreover, NfL plasma levels have been recently associated in Parkinson’s Disease in patients with depressive symptoms (48). Despite this pointing evidence, we have not found any statistical correlation between subjective scales measuring depression severity (HDRS), anhedonia (SAAS), general cognitive state (MMSE) or episodic memory (FCSRT) with NfL plasma levels in MDD patients. Conversely, previous studies found a significant and positive correlation between NfL levels and the HAM-D6 scale (28). In terms of cognition, we have only found in the literature a significant correlation between NfL levels and poorer performance in executive function task measured with the Wisconsin Card Sorting Test (WCST) in MDD patients (24).

Finally, there is still need for evidence on how pharmacological treatment with antidepressants might affect NfL peripheral levels in MDD patients and whether alterations in NfL levels could represent a predictive marker in MDD patients at risk of developing future neurodegenerative disorders.

## 5 Limitations and future perspectives

The present study had to face the following limitations. First, the cross-sectional nature of our study has not allowed us to explore prospective changes in NfL levels in MDD patients after a controlled period with antidepressant treatment. Second, we did not have access to more potential confounding parameters such as body mass index. Third, we could not measure subjective scales regarding cognitive domains such as MMSE and FCSRT in the HC group to establish a proper comparison between MDD and HC and also to check if cognitive differences may be occurring despite NfL alterations or if they both cognitive alterations may act as predictors. Fourth, the sample size of our study is relatively small and future research can be done to improve this statistical aspect in order to extract conclusions that are more robust. However, our primary goal was just to explore NfL peripheral levels in a Spanish cohort of patients suffering from MDD to test whether there could be any evidence of axonal damage in relation to a major depression diagnosis. Finally, we suggest to explore NfL longitudinal changes in response to antidepressant treatment in MDD patients to evaluate pharmacological effects as a potential mediator in NfL alterations.

## Data Availability

The raw data supporting the conclusions of this article will be made available by the authors, without undue reservation.
